# Enhancing Engagement, Practice Integration, and Skill Learning in Mobile Technology–Delivered Interventions Using Human Support: Randomized Controlled Trial With Depressed College Students

**DOI:** 10.2196/56963

**Published:** 2025-08-25

**Authors:** Colleen S Conley, Brynn M DeLorenzo, Carol H Gonzales, Ian J Kahrilas, Jennifer Duffecy, Rebecca L Silton

**Affiliations:** 1 Psychology Department Loyola University Chicago Chicago, IL United States; 2 Department of Psychiatry Massachusetts General Hospital Boston, MA United States; 3 Saint Louis Children's Hospital St. Louis, MO United States; 4 Kythera Labs Franklin, TN United States; 5 Mood and Anxiety Disorders Program Department of Psychiatry University of Illinois Chicago Chicago, IL United States

**Keywords:** mobile app, digital intervention, human support, engagement, skill integration, skill learning, mindfulness, depression

## Abstract

**Background:**

Among evidence-based mobile technology–delivered interventions (mTDIs), mindfulness apps such as Headspace have demonstrated numerous benefits. These benefits are particularly important for college students, who continue to face high rates of depression and psychological distress that are paired with insufficient mental health services to meet these needs. mTDIs offer scalable solutions to ameliorating mental health symptoms and may be able to help address this gap in limited access to mental health services for all populations. While mTDIs have great promise for maximizing reach, their utility can be hamstrung by low rates of user engagement and uptake. Thus, this study implemented 2 human support enhancements designed to boost user in-app engagement, practice integration into daily life (ie, sustainability), and app-related skill learning (ie, perceived benefits) in a sample of college students with depression who were granted full access to an mTDI (Headspace).

**Objective:**

This randomized controlled trial evaluated the impact of two human support enhancements—(1) a one-time face-to-face orientation with or without (2) placement in a peer supportive accountability group—on self-reported and objectively captured mTDI engagement, practice integration, and skill learning among a sample of college students with depression.

**Methods:**

Participants (n=123) authorized access to their recorded app use data, provided by Headspace. In addition, at the midpoint (1 mo), postintervention (2 mo), and follow-up (3 mo) assessments, participants self-reported on the extent to which they had used the app, how likely they were to continue using the app and related skills in the future, and the extent to which they learned skills and practiced these skills in their daily lives.

**Results:**

Compared to participants who were simply given access to the app (37/123, 30.1%) without these enhancements, those who attended the orientation (86/123, 69.9%), regardless of additional random allocation to the peer supportive accountability group (48/123, 39%), demonstrated significantly greater mTDI engagement (ie, more minutes meditated [*F*_2,117_=11.20; *P*<.001] and more sessions overall [*F*_2,117_=15.00; *P*<.001]) and rated more favorably multiple aspects of practice integration (ie, more everyday mindfulness practice [*F*_2,72_=6.20; *P*=.003] and greater likelihood of future mindfulness [*F*_2,71_=7.42; *P*<.001]) and skill learning (ie, learning about mindfulness [*F*_2,73_=6.02; *P*=.004], learning mindfulness skills [*F*_2,72_=11.01; *P*<.001], and an increased awareness of thoughts and feelings [*F*_2,73_=6.05; *P*=.004]), indicating potential implications for amplifying the benefits of mTDIs through increased user engagement.

**Conclusions:**

The results of this study illustrate that an initial face-to-face orientation boosts mTDI engagement, enhances the integration of intervention skills into everyday life, and increases learning. Future work is needed to determine the active ingredients of the orientation as well as to narrow in on the optimal implementation of supportive accountability that might drive increased levels of engagement and the associated positive intervention benefits.

**Trial Registration:**

Open Science Foundation (OSF) 3trzk; https://osf.io/3trzk

## Introduction

### Background

Evidence-based technology-delivered interventions (TDIs), including mobile apps that promote wellness and mental health (mobile TDIs; mTDIs), have demonstrated great promise for improving emotional well-being and reducing psychological distress across various population samples, including college students [1,2]. Mindfulness apps in particular have been effective in reducing depression, anxiety, and stress and improving psychological well-being and life satisfaction [3]. Importantly, mindfulness mTDIs also promote the development of related skills, with participants reporting significant improvements in areas such as awareness, acceptance, nonjudgmental attitude, and focus on the present moment [4,5]. Thus, these tools offer an effective and accessible way to address the large treatment gap that exists between individuals who need mental health treatment and those who are receiving it, a discrepancy that is especially pronounced for college students with depression [6].

Beyond their clinical utility, mTDIs have broad appeal, especially among college students, who seem to prefer the convenience, immediacy, and perceived confidentiality [7] paired with the added benefit of 24-hour access, including availability after in-person clinic hours [1]. Although there are hundreds of mTDIs designed to address mental health problems, and these numbers are growing rapidly, many of these apps are not evidence-based [8,9]. Furthermore, clinical science grapples with translating the benefits often seen when evaluating mTDIs through research trials [10] into best practices for promoting real-world applications, often marked by low uptake and engagement [11]. Interventions might work well for a subset of people who engage with them but not for those who fall short of engagement or adherence standards [4]. Indeed, users cannot benefit from the behavioral changes facilitated by mTDIs if they do not engage with such programs and learn the necessary skills. To harness the potential benefits of mTDIs, it is an urgent priority to identify strategies and supports for fostering sustained engagement and integration into daily life so that users can ultimately learn and implement skills for well-being.

Although mTDIs are typically designed to be self-guided, they can be integrated with human support features to enhance user engagement and thus benefits. This study is part of a larger randomized controlled trial (RCT) that established the efficacy—specifically psychosocial benefits—of a mindfulness-based mTDI for college students experiencing depressive symptoms. Building on these findings, this study examines whether 2 human support enhancements—a face-to-face orientation and placement in small peer supportive accountability groups—(1) boosted *engagement* with the mindfulness-based mTDI, (2) cultivated increased *practice integration* into daily life, and (3) enhanced users’ self-reported *skill learning*.

### Engagement With Mobile Interventions: Challenges and Opportunities

Although mTDIs have great potential to deliver effective treatments to a broad population, such programs are marked by low rates of both uptake and sustained user engagement, particularly for self-guided treatments that involve lower levels of structure and prescriptive guidance [12,13]. Such digital approaches require users to be self-motivated to initiate and sustain their use independently [14]. Furthermore, the mental health symptoms driving users to seek out mTDIs, such as depressive symptoms (eg, reduced interest, energy, and concentration), may themselves interfere with mTDI engagement [15]. Unpacking patterns of user engagement along with studying intervention design strategies to boost and maintain engagement are critical steps for advancing the efficacy of evidence-based mTDI interventions.

Engagement has been defined as “a state of energy investment involving physical, affective, and cognitive energies directed toward a focal stimulus or task” [16,17]. For the purposes of this study, engagement is measured behaviorally via the number of use minutes and completed sessions recorded by the mTDI software. While there is ongoing debate regarding how to best define and measure engagement [18], tabulating app use data (including duration and frequency) remains one of the most common methods [19]. User data from mindfulness-based mTDIs generally show engagement rates that start high but gradually reduce over time, with precipitous drops within the first week of uptake [11]. Of concern, engagement is particularly low during self-guided follow-up study periods [20,21]; for example, Flett et al [4] found that college students used a mindfulness-based mTDI almost daily in the primary 10 days of the study, but less than half had *any* use in the subsequent “30-day extended use period”. This sentiment is reflected in conversations with college student app users as well: despite almost 75% reporting some benefit from mTDIs, the same proportion engaged with them weekly or less frequently [7]. Such research highlights the discrepancy between interest in, and engagement with, mTDIs.

While emerging research in the area of digital mental health frequently recommends the integration of human support to enhance mTDI engagement, there remain many questions related to what type of support is needed; for example, there is great variability in the provider (eg, peers, unlicensed professionals, and licensed clinicians), the modality (eg, in-person contact, video visits, telephone calls, and text-based messaging), and the frequency of support provided. Further complicating this issue is that few studies provide a detailed description of the human support that was delivered or examine the role of such support in their study outcomes, making it difficult to draw larger conclusions across studies [22]. This study aims to address this gap by specifically examining the impact of 2 human support features—participation in a preintervention orientation session and placement in a peer supportive accountability group—on mTDI engagement, practice integration beyond the app, and skill learning.

### Peer Supportive Accountability

Supportive accountability is a theoretical model that seeks to explain how human support can improve engagement with mTDIs [14]. The model posits that a user is likely to engage more with an mTDI when they are accountable to another person. Accountability includes knowing that actions or inactions with the intervention will be observed, and behavioral choices will have to be justified to someone. Ideally, the person a user is being accountable to will be viewed as legitimate and with beneficial knowledge to offer, and therefore the accountability process is viewed as supportive and noncoercive. A systematic review of 208 studies examining user engagement with mTDIs did not explicitly test the supportive accountability model, but it found that guided or supported interventions had higher engagement than self-guided interventions and that being able to connect with others through the mTDI increased engagement [15].

The supportive accountability theory has been tested in a number of formats, including telephone coaching from clinicians and peers as well as through digital formats [14,23]. Coaching delivered by peers is as effective at improving engagement as coaching delivered by licensed clinicians [24]. Peer-delivered support typically has been offered through embedded message boards that include components of visible goal setting and completion as well as methods to provide support and communication to increase goal completion (eg, “likes” and comments). Several studies across different populations have demonstrated increased mTDI use by participants with access to peer support features compared to completely self-guided participants [25-27]. Even in trials with no differences in engagement between groups or no self-guided comparison group, participants tend to rate peer supportive accountability as positive and helpful [24,28-30]. A systematic review of 24 peer-supported digital mental health interventions concluded that using peer support to deliver or supplement mTDIs is feasible, acceptable, and effective at improving engagement and psychological functioning [31]. Ultimately, incorporating supportive accountability features into mTDIs for depression has the potential to improve both engagement and mental health outcomes for a wide array of users.

### Preintervention Orientation

Research has less commonly explored the ways in which one-time support interactions, such as an orientation to a TDI, might enhance engagement. Psychotherapy research has explored the benefits of engagement sessions, typically using motivational interviewing (MI) tools, to enhance engagement with traditional mental health services [32,33]. More recently, MI has been incorporated into TDIs as a potential engagement strategy. Such techniques allow users to interact with programs in a more intentional manner by setting expectations and goals for program use, encouraging the use of problem-solving and other skills, and normalizing challenges [34]. More specifically, using MI to support TDI users as they prepare for, and enact, behavioral changes is particularly encouraged to support program engagement [34]. Brief motivational interventions, such as through SMS text messages or telephone calls, are viewed positively by TDI users, promote program engagement, and even enhance initial program outcomes [35-38]. Similarly, Linardon and Fuller-Tyszkiewicz [13] found increased engagement for trials that had at least 1 opportunity for contact with the researchers (either a telephone interview or an in-person interview before enrolling) compared to trials where participants could enroll in a study on the web without contacting a researcher.

Some research has targeted motivation before users engage with the program, such as through a synchronous (eg, face-to-face or telephone) orientation session with research staff or through a module within the TDI. While some studies seem to incorporate such techniques into their methodological design [29,39], few have provided details about the content of these sessions or explicitly investigated their impact on engagement or other outcomes. Encouragingly, researchers have begun to recognize that such techniques may serve as an active component of treatment that can be investigated in and of itself; for example, Bur et al [40] conducted an RCT of a web-based self-help program for depression, exploring the effects on user engagement of different types of support, including elements with human contact (personal guidance and a preintervention diagnostic interview) and those without human contact (automated reminders and a preintervention MI module). At the end of the intervention, participants who received human support in the form of individualized guidance demonstrated both greater reductions in depressive symptoms and greater treatment adherence compared to the other support conditions [40]. Beyond building motivation for engagement, these preprogram touchpoints can help users navigate the technology of a new app as well as the large amounts of content that many TDIs provide, both of which can be overwhelming, particularly when users are also contending with mental health challenges such as depression [8]. Overall, more research is needed to better understand the role that preprogram interventions (eg, introductory, orientation, or engagement sessions) may have and their effects on subsequent program engagement and associated benefits.

### Self-Guided Practice for Lasting Benefits

As with skills-based face-to-face treatments, the goal of mTDIs is to help users learn and practice skills so that they become integrated into their daily lives over time. Notably, the completion of out-of-session exercises is uniquely linked to symptom improvement and treatment outcome across disorders [41]. In a similar vein, Schlosser et al [42] distinguished between TDI active use (eg, tasks or skill practices completed) and passive use (eg, time on the app that was not spent meaningfully engaging with other users or task completion) and found that only active use was linked to symptom improvement. This signals the critical importance of assessing not only objective engagement counts (eg, TDI use minutes and completed sessions) but also broader elements such as practice integration and skill learning.

### Study Aims and Hypotheses

In a sample of college students with elevated depression, we examined the benefits of two mTDI enhancements—(1) a face-to-face orientation with or without (2) placement in a peer supportive accountability group—on three sets of outcomes: (1) mTDI engagement, (2) practice integration, and (3) skill learning. For the first set of outcomes—mTDI engagement—we hypothesized that those who were randomly allocated to receive the enhancements would demonstrate greater mTDI engagement—in terms of (1) mindfulness minutes and (2) total sessions completed—compared to those who were simply given access to the app without either of these enhancements. We examined whether participants in the peer supportive accountability group experienced incremental benefits in engagement, beyond those of the orientation. In an exploratory fashion, we examined whether there were differences among the 3 intervention groups on 2 other sets of self-report outcomes: For the second set of outcomes, practice integration, we specifically examined (3) “everyday mindfulness” and the likelihood of engaging in future mindfulness practices, whether (4) using the app, (5) engaging in mindfulness exercises on one’s own, or (6) generally being more mindful in daily life. For the third set of outcomes, skill learning, we specifically examined (7) learning about mindfulness as a concept and practice, (8) learning mindfulness skills, and (9) being aware of one’s thoughts and feelings.

## Methods

### Ethical Considerations

This RCT (registered on OSF before the start of analyses) was approved by the Loyola University Chicago Institutional Review Board (project number 2330), and all participants provided informed consent. Minimal identifying data were collected, and identifiers were removed from the dataset for analysis. Confidential (ie, identifying) data were stored on a university-supported, password-protected network drive and in locked filing cabinets to which only the research team had access. Participants were compensated monetarily (up to US $70) or with participant pool course credits (up to 10 credits) for the completion of study sessions.

### Recruitment

Over 8 successive semesters, we recruited undergraduate students from a midsized Midwestern university using a multipronged voluntary response sampling strategy, including the psychology participant pool; cross-campus listserve emails; and flyers seeking students who self-identified as “down, sad, or distressed.” Recruitment occurred at the beginning of each semester between 2017 and 2021. Eligible participants endorsed clinically significant levels of depressive symptoms as indicated by a score of ≥10 on a brief web-based screening using the Patient Health Questionnaire-8 (PHQ-8), which omits the suicidality item [43,44]. Individuals were excluded if they were engaged in psychotherapy at the start of the study, had regular practice of mindfulness or consistent use of the Headspace app within the past 6 months, or reported that they were unwilling to join the peer supportive accountability group if randomly allocated to this condition. As the broader project included electroencephalography assessments, participants were also excluded if they had a history of neurological conditions or head trauma (eg, concussions or seizures). Our sample size (10-27 students each semester) was determined by the number of participants who met study criteria each semester, paired with a consideration of resource constraints (ie, time, space, and funding) [45].

### Intervention Groups and Procedures

#### Overview

Participants were randomly allocated to 3 nested groups: app as usual (App), app+orientation (App+O), and app+orientation and peer supportive accountability (App+OPS). Specifically, all participants received the base intervention of access to the app (App), all App+O and App+OPS participants (but not App participants) attended an orientation, and only the App+OPS participants were invited to the peer supportive accountability features (as described in the next subsection).

An unequal allocation procedure was used for randomization to ensure that a consistent set of 6 participants was assigned to each peer supportive accountability group (totaling to 8 groups over 8 semesters). Specifically, we performed a manual blind drawing to randomly allocate 6 participants to each App+OPS group and to randomly and evenly split the remaining participants among the App+O and App groups. This procedure yielded a final allocation ratio of 1.26:1.00:0.97. Participants completed 4 web-based self-report surveys providing demographic information before the intervention (within 2 wk before code activation) and assessing their experiences with the intervention at midpoint (1 mo after code activation), after the intervention (2 mo after code activation), and at 1-month follow-up (3 mo after code activation).

#### Intervention: Mobile Mindfulness App (Headspace)

Intervention participants received a 3-month code to access all content on Headspace [46], a web-based application and mobile app that delivers brief, guided mindfulness exercises as well as some additional wellness-related content (eg, “soundscapes,” “sleepcasts,” and “focus music”). The program includes courses that typically include 10 to 30 sessions that follow a particular theme (eg, handling sadness and managing anxiety). Headspace also includes single mindfulness meditation sessions, some of which are intended to be used in a specific situation (eg, difficult conversations) or as guided mindful activities (eg, mindful eating and mindful walking). Users can customize the length of most sessions, ranging from shorter (1-5 min) to longer (10-30 min) options. Although participants were free to access any of the Headspace content, they were encouraged to engage with mindfulness exercises, particularly the “Basics” courses teaching foundational skills on mindfulness and meditation and the mental health–related meditations, such as those focused on psychological distress (eg, sadness, stress, anxiety, and “SOS” sessions) and positive well-being (eg, happiness and self-esteem).

#### Intervention: Orientation

The App+O and App+OPS groups (but not the App group) attended a 90-minute group orientation session wherein the first 3 authors reviewed study procedures, briefly described the principles and benefits of mindfulness, oriented participants to app features and content, helped them to activate their accounts with access codes, and provided recommendations for mTDI engagement. Although participants were encouraged to use the app as frequently and consistently as possible (ie, daily) and to use its mental health–focused content, no specific requirements were given, and use was ultimately self-guided. As recommended for mTDI engagement [34], elements of MI [47] were interspersed throughout the orientation session; for example, participants were encouraged to identify specific goals for their mTDI engagement and mindfulness practice, to consider their expectations and motivations for engaging in the program, to predict barriers to app use and engage in related problem-solving, and to reflect on approaches that have and have not been successful for them in the past in terms of establishing a new habit. Broader techniques such as eliciting participant thoughts and ideas, providing reflections, and using open-ended questions were also incorporated. At this point in the orientation, participants in the App+O group were dismissed, and for the duration of the study, they did not have additional contact with other study participants or the research staff beyond communication related to research assessments and compensation.

#### Intervention: Peer Supportive Accountability

Participants randomly allocated to the App+OPS group attended the aforementioned group orientation and stayed for an additional 20 to 30 minutes to review the rationale and procedures for the peer supportive accountability features. Researchers provided an overview of supportive accountability as well as the benefits of social sharing and of giving and receiving support. In cohorts of 6, App+OPS participants added each other as Headspace “buddies,” which allowed them to share their progress. As time allowed, participants also set individual and group goals for their mindfulness practice (eg, daily use of the app) and for engagement with the peer supportive accountability group (eg, weekly posting in the web-based forum).

Participants in the App+OPS condition joined a private, closed Facebook group that served as a web-based forum. Participants were encouraged to post in the forum about their own successes and struggles with mindfulness practice; pose questions to the group; share information; and provide motivation, accountability, and support to other group members. Three to 5 times per week, research staff posted Headspace “mindful moments” (eg, “Imagine a world in which we witness thoughts without becoming them and experience feelings without being overwhelmed by them”), along with prompts encouraging participants to share their experiences with mindfulness and the app (eg, “What do you imagine? How would things be different?”). Twice per week, research staff posted user statistics, at the individual and group level (ie, number of sessions and minutes completed, session content, and days of use), along with an email digest that included the same user statistics and quotes from the web-based forum, examples of research findings demonstrating the benefits of mindfulness, and links to the web-based group and to Headspace.

In the third semester (and cohort) of the study, a face-to-face component was added to the App+OPS condition based on participant feedback. Participants met for 3 in-person group sessions approximately every other week (ie, 2, 4, and 6 wk after the orientation) for 45 to 60 minutes. Sessions did not include mindfulness exercises but rather focused on peer supportive accountability, following a general structure wherein research staff gave a brief introduction and reminder of the purpose of the group sessions, including any goals that were set in the previous session. With minimal direction from research staff, participants openly discussed their experiences with mindfulness (eg, barriers and successes), checked in with one another about progress, and fostered a sense of connectedness. During each meeting, a slide with suggested discussion topics was displayed for the group. At the end, research staff highlighted themes from the discussion and prompted participants to consider setting group and individual goals.

### Measures

#### Social Identities

During the preintervention assessment, participants responded to a series of questions about different aspects of social identities, including age, racial and ethnic identity, gender, and sexual identity.

#### Engagement With mTDI

With participant consent, Headspace provided user data for each session, including date, time of day, module (eg, “Basics”), session number within the module (eg, session 1), and duration in minutes. Researchers calculated each participant’s total minutes of mindfulness practice (mindfulness minutes) and total sessions of any type (total sessions). All meditation sessions (the vast majority of content in the app) as well as mindful activities (eg, guided mindful walking) were considered mindfulness practice. Sessions of advice, music (“focus music” and “sleep music”), and ambient noise and sounds (“sleepcasts” and “soundscapes”) were not included in the totals of mindfulness minutes; however, they were included in total sessions to capture participants’ broader engagement with the app.

#### Practice Integration: Self-Reported Mindfulness Practices and Likely Future Mindfulness Practice

To assess participants’ practice integration or the use of mindfulness practice outside of the app, at the midpoint, postintervention, and 1-month follow-up assessments, participants were asked about (1) their self-reported frequency of “nonguided, everyday mindfulness” (ie, self-guided practice) since starting the intervention. Participants rated how often they had practiced this on a Likert scale from 1 (“none: not at all”) to 5 (“a lot: daily practice”). Participants were also asked how likely they were, *after* the study, to (2) use Headspace (not considering cost), (3) perform mindfulness exercises on their own, and (4) be more mindful in their everyday life. Participants rated each of these items on a scale ranging from 1 (“not likely”) to 5 (“extremely likely”).

#### Skill Learning

Finally, at the midpoint, postintervention, and 1-month follow-up assessments, participants were asked about their perceptions of the intervention and its benefits, adapting some items from the mindfulness intervention social validity questionnaire [48]. Participants were asked to consider their overall experience with the program and report the extent to which they felt they had, through the program, learned about mindfulness, learned mindfulness skills, and gained awareness of their thoughts and feelings. Participants rated each item on a scale ranging from 1 (“not at all true”) to 5 (“extremely true”).

### Data Analysis Plan

In examining group effects, we used an intent-to-treat strategy, including all participants who were randomly allocated to 1 of the 3 intervention groups and for whom we had relevant data. For example, of the 120 participants with valid engagement data, a small subset (10/120, 8.3%; all in the App group) did not engage with the app; following an intent-to-treat approach, these participants were included in analyses. Furthermore, all App+OPS participants with valid data were included in the analyses, regardless of their level of engagement in the web-based forum or attendance at group meetings. All statistical analyses were conducted using SPSS software (version 28.0; IBM Corp) [49]. We report observed effect sizes in the results. Missing data were handled via case-wise deletion for each specific analysis.

To examine overall differences among the 3 groups across 3 time points (midpoint, postintervention, and 1-mo follow-up), we conducted group×time ANOVAs and interpreted the group effects. For outcomes with an overall effect of group (human support condition), we examined pairwise contrasts to identify any differential impact of the orientation alone versus the orientation plus peer supportive accountability.

Accounting for the effect of running multiple tests on type I error, we calculated a Bonferroni correction by dividing .05 by the total number of individual ANOVAs and consider both the standard significance threshold (*P*<.05) as well as the Bonferroni-adjusted significance threshold (*P*<.0055) in evaluating statistical significance. We also report effect sizes [50]. Specifically, we examined partial eta-squared values using the standards proposed by Cohen [51] for small (η_p_^2^≥0.01), medium (η_p_^2^≥0.06), and large (η_p_^2^≥0.14) effects. We evaluated the distribution (skewness) of outcome variables at each time point, and we also ran Kolmogorov-Smirnov tests for each variable (Multimedia Appendix 2).

## Results

### Participant Characteristics

**Figure 1:**
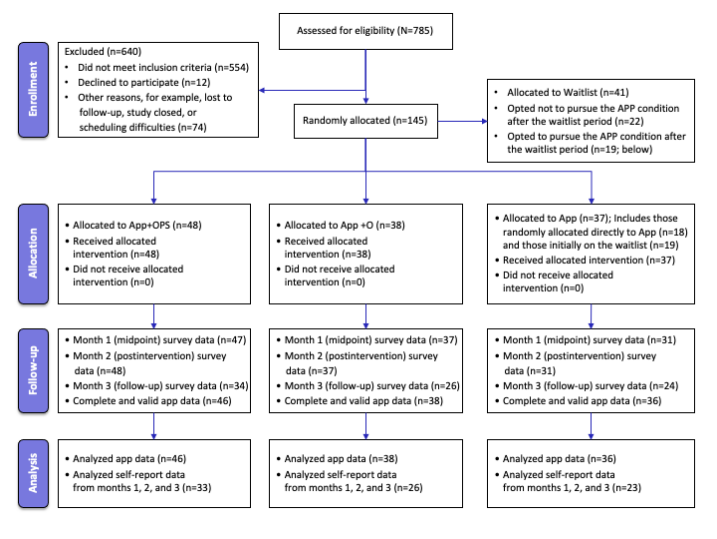
CONSORT (Consolidated Standards of Reporting Trials) flow diagram. App: app as usual; App+O: app+orientation; App+OPS: app+orientation and peer supportive accountability.

Participant recruitment yielded 123 undergraduate students (age: mean 19.1, SD 1.5 y), with an average PHQ-8 screening score of 14.1 (SD 3.4; range 10-22), randomly allocated to use the app as follows: 30.1% (37/123) App participants, 30.9% (38/123) App+O participants, and 39% (48/123) App+OPS participants (refer to Figure 1 for the CONSORT [Consolidated Standards of Reporting Trials] flow diagram; the results are presented according to the CONSORT-EHEALTH [Consolidated Standards of Reporting Trials of Electronic and Mobile Health Applications and Online Telehealth] checklist [Multimedia Appendix 1]). Of note, of the 37 participants in the App group, 51% (19/37) first completed a waitlist period, and these participants did not differ from the other 49% (18/37) of App participants who activated their Headspace access code at the same time as the rest of their cohort or from App+O or App+OPS participants on baseline depression (*F*_3,120_=0.30; *P*=.83). Table 1 presents baseline demographic characteristics for the full sample and for participants randomly allocated to the 3 study groups, reflecting a higher proportion of women attending college and endorsing depression, but demonstrating no baseline group differences (refer to footnote a below Table 1).

A few details shown in the CONSORT flow diagram are worth elaborating on. Three participants (n=1 in the App group and n=2 in the App+OPS group) did not have valid app-recorded engagement data due to data recording errors or access code malfunctions; therefore, they were excluded from analyses examining objective mTDI engagement. In addition, there were some missing data from surveys, as noted in the CONSORT flow diagram. Notably, participants who completed surveys at all 3 time points (82/123, 66.7%) did not differ from those who completed surveys at only 1 or 2 time points (41/123, 33.3%) in gender (*χ*^2^_2_=0.1; *P*=.96), sexual identity (*χ*^2^_4_=2.9; *P*=.58), racial and ethnic identity (*χ*^2^_5_=2.9; *P*=.71), or baseline depression scores (t_120_=0.81; *P*=.42) but were more likely to be younger in age (t_121_=2.77; *P*=.006) and a first-year student (*χ*^2^_4_=10.5; *P*=.03).

**Table 1:** Baseline demographic characteristics for the full sample and for participants randomly allocated to the 3 study groups^a^.

			Total sample (n=123)		App+OPS^b^ (n=48)		App+O^c^ (n=38)		App^d^ (n=37)
Age (y), mean (SD)			19.1 (1.5)		19.2 (1.3)		19.2 (1.8)		18.9 (1.1)
**Gender, n (%)**									
	Woman	113 (91.9)		44 (1.7)		34 (89.5)		35 (4.6)	
	Man	7 (5.7)		4 (8.3)		1 (2.6)		2 (5.4)	
	Self-identified response (eg, nonbinary or transgender)	3 (2.4)		0 (0)		3 (7.9)		0 (0)	
**Sexual identity, n (%)**									
	Bisexual	23 (18.7)		5 (10.4)		9 (23.7)		9 (24.3)	
	Gay	4 (3.3)		3 (6.3)		1 (2.6)		0 (0)	
	Heterosexual	89 (72.4)		37 (77.1)		26 (68.4)		26 (70.3)	
	Lesbian	1 (0.8)		0 (0)		0 (0)		1 (2.7)	
	Self-identified response (eg, asexual, pansexual, or queer)	6 (4.9)		3 (6.3)		2 (5.3)		1 (2.7)	
**Racial and ethnic identity, n (%)^e^**									
	African American or Black	1 (0.8)		1 (2.1)		0 (0)		0 (0)	
	Asian or Asian American	19 (15.4)		5 (10.4)		6 (15.8)		8 (21.6)	
	Hispanic or Latiné	19 (15.4)		5 (10.4)		7 (18.4)		7 (18.9)	
	Native American or Pacific Islander	1 (0.8)		0 (0)		0 (0)		1 (2.7)	
	Non-Hispanic White	69 (56.1)		28 (58.3)		21 (55.3)		20 (54.1)	
	Multiracial or self-identified (eg, Middle Eastern)	14 (11.4)		9 (18.8)		4 (10.5)		1 (2.7)	
**Year in school, n (%)**									
	First-year undergraduate	69 (56.1)		26 (54.2)		20 (52.6)		23 (62.2)	
	Second-year undergraduate	30 (24.4)		13 (27.1)		12 (31.6)		5 (13.5)	
	Third-year undergraduate	12 (9.8)		4 (8.3)		2 (5.3)		6 (16.2)	
	Fourth-year undergraduate	10 (8.1)		3 (6.3)		4 (10.5)		3 (8.1)	
	Other (eg, accelerated nursing student)	2 (1.6)		2 (4.2)		0 (0)		0 (0)	

^a^There were no significant differences across groups in age (*F*_2,120_=0.35; *P*=.71) or other demographic data: gender (*χ*^2^_4_=8.0; *P*=.09), sexual identity (*χ*^2^_8_=8.6; *P*=.38), racial and ethnic identity (*χ*^2^_10_=11.7; *P*=.30), or year in school (*χ*^2^_8_=9.2; *P*=.33).

^b^App+OPS: app+orientation and peer supportive accountability.

^c^App+O: app+orientation.

^d^App: app as usual.

^e^Racial and ethnic identity categories were based on National Institutes of Health reporting guidelines from 2017, which reflected the categories identified by the US Office of Management and Budget standards for the classification of federal data on racial and ethnic identity.

### Impact of Human Support Enhancements

The following subsections present the results for the impact of 2 human support enhancements (a face-to-face orientation with or without placement in a peer supportive accountability group) on the 3 categories of outcomes: (1) engagement with the mTDI (minutes and sessions), (2) practice integration (eg, everyday mindfulness practice beyond the mTDI), and (3) skill learning (eg, self-reported awareness, learning, and skills).

#### Engagement With mTDI

**Figure 2:**
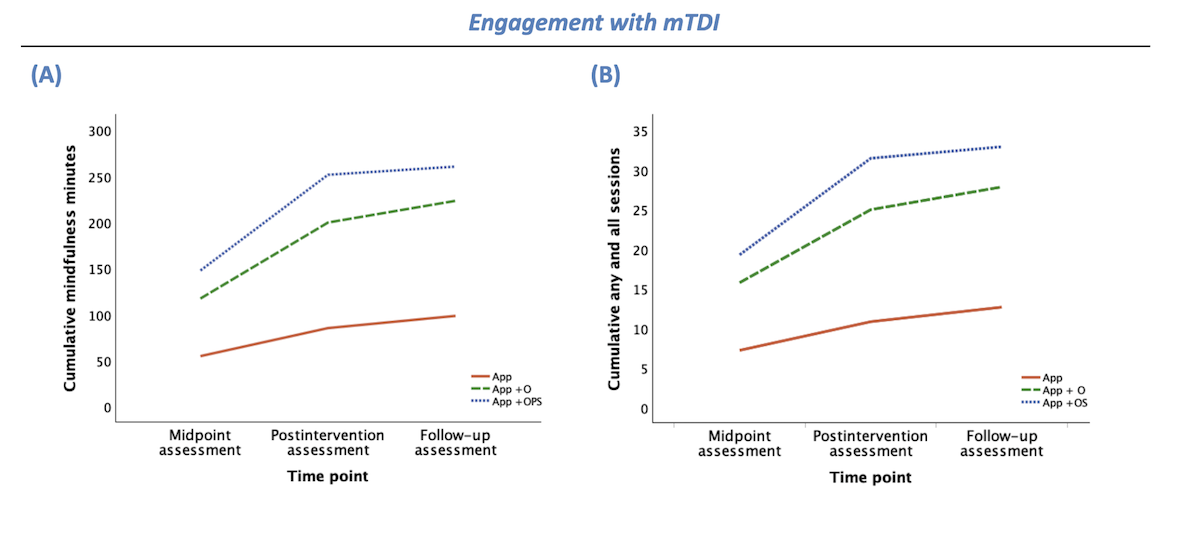
Engagement with mTDI group means by time point (midpoint, postintervention, and 1-mo follow-up assessments) for participants randomly allocated to use the app without human support enhancement (app as usual [APP]; solid orange line), participants randomly allocated to attend a 1-time orientation (app+orientation [APP+O]; dashed green line), and participants randomly allocated to attend the orientation and be placed in a peer supportive accountability group (app+orientation and peer support [APP+OPS]; dotted blue line). Engagement outcomes include objectively captured: (A) cumulative mindfulness minutes, (B) cumulative any and all sessions. The y-axes reflect totals. mTDI: mobile technology–delivered intervention.

**Figure 3:**
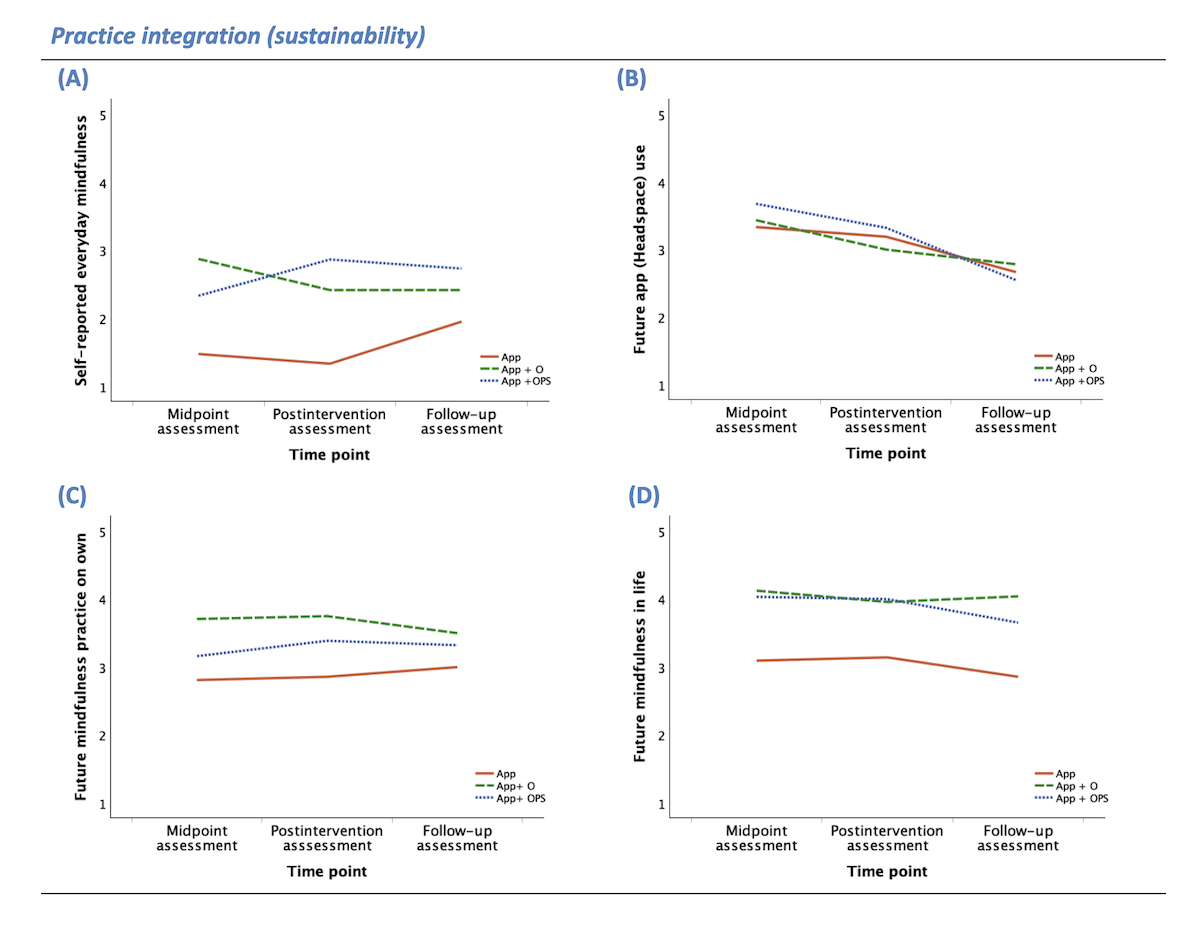
Practice integration (sustainability) group means by time point (midpoint, postintervention, and 1-mo follow-up assessments) for participants randomly allocated to use the app without human support enhancement (app as usual [APP]; solid orange line), participants randomly allocated to attend a 1-time orientation (app+orientation [APP+O]; dashed green line), and participants randomly allocated to attend the orientation and be placed in a peer supportive accountability group (app+orientation and peer support [APP+OPS]; dotted blue line). Practice integration outcomes include self-reported: (A) everyday mindfulness, (B) future app (Headspace) use, (C) future mindfulness practice on own, (D) future mindfulness in life. The y-axes reflect scale means (range 1-5).

**Figure 4:**
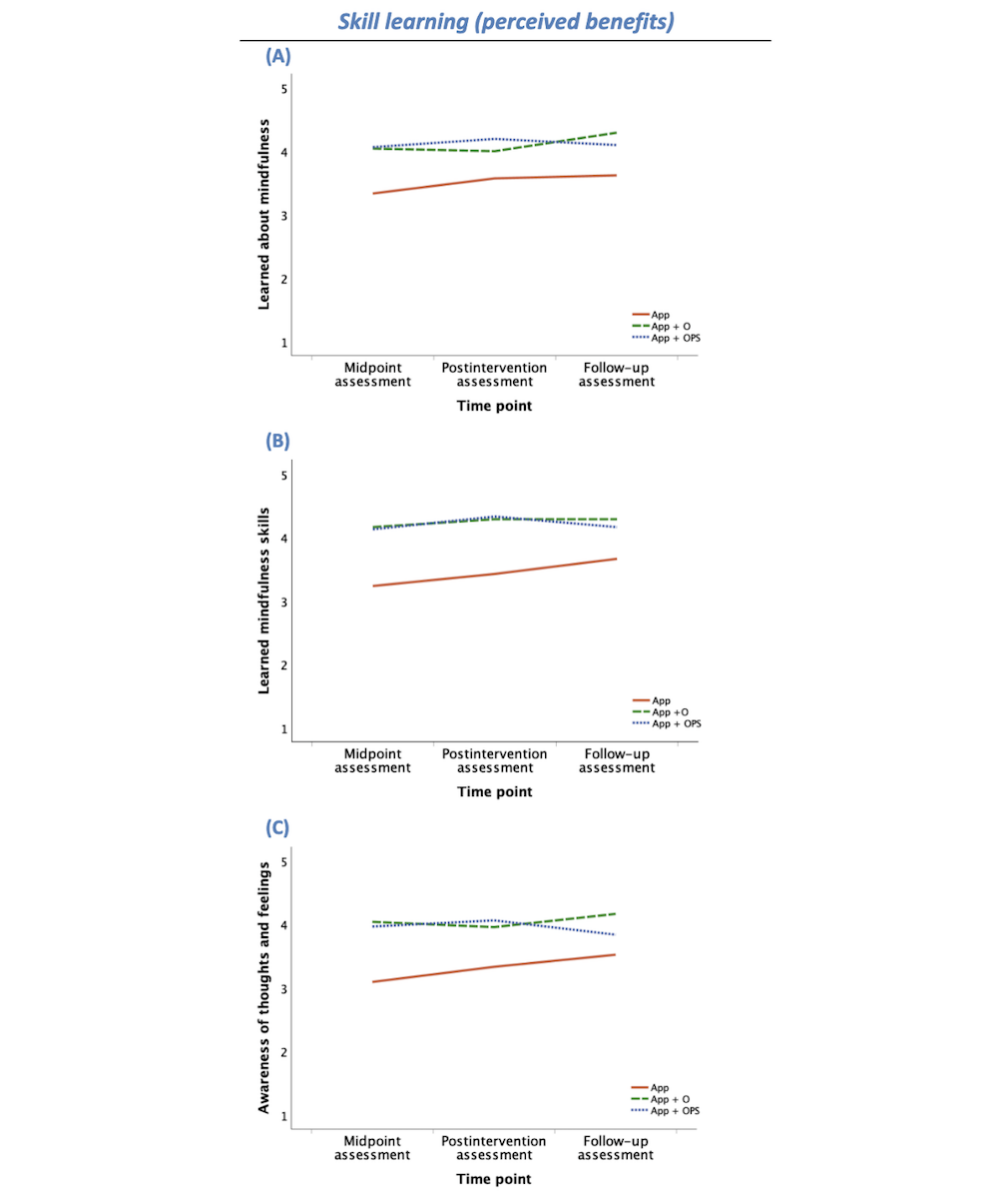
Skill learning (perceived benefits) group means by time point (midpoint, postintervention, and 1-mo follow-up assessments) for participants randomly allocated to use the app without human support enhancement (app as usual [APP]; solid orange line), participants randomly allocated to attend a 1-time orientation (app+orientation [APP+O]; dashed green line), and participants randomly allocated to attend the orientation and be placed in a peer supportive accountability group (app+orientation and peer support [APP+OPS]; dotted blue line). Skill learning outcomes include self-reported: (A) learning about mindfulness, (B) learning mindfulness skills, and (C) awareness of thoughts and feelings. The y-axes reflect scale means (range 1-5).

Table 2 presents group means and SDs and summarizes group effects (*F*, η_p_^2^, and *P* values) across the trial for each outcome. In addition, Figures 2-4 depict group means over time for each outcome. As shown in Table 2, there was an overall effect of group (human support condition) on cumulative mindfulness minutes (*F*_2,117_=11.20, η_p_^2^=0.16; *P*=.001). Pairwise comparisons indicate that App participants had significantly lower mindfulness minutes than App+O (*P*=.005) and App+OPS (*P*<.001) participants, but mindfulness minutes did not significantly differ between App+O and App+OPS groups (*P*=.55; Figure 2A).

**Table 2:** Group means and SDs by time point—midpoint assessment (1 month after initiating app), postintervention assessment (2 months after initiating the app), and 1-month follow-up assessment (3 months after initiating the app, and 1 month after the intervention)—and group effects comparing participants randomly assigned to use a mindfulness app as usual (App; n=36), app+orientation (App+O; n=38), and app+orientation and peer supportive accountability (App+OPS; n=46).

Outcomes^a^				Group means (SDs)							ANOVA group effects						
				Midpoint		Postintervention		Follow-up		*F* test (*df*)			η_p_^2^			*P* value	
**Engagement with mobile technology–delivered intervention**																	
	**Cumulative mindfulness minutes**										11.20 (2, 117)			0.161			<.*001*
		App (n=36)^b^	55.0 (87.2)		85.4 (143.9)		98.6 (162.8)										
		App+O (n=38)^c^	117.4 (97.6)		199.9 (181.0)		223.4 (212.3)										
		App+OPS (n=46)^c^	147.8 (80.2)		251.7 (132.6)		260.5 (138.2)										
	**Cumulative** **any and all** **sessions**										15.00 (2, 117)			0.204			<.*001*
		App (n=36)^b^	7.22 (9.58)		10.83 (15.35)		12.67 (17.86)										
		App+O (n=38)^c^	15.76 (10.11)		24.97 (17.31)		27.84 (20.95)										
		App+OPS (n=46)^c^	19.28 (9.48)		31.46 (16.28)		32.91 (17.43)										
**Practice integration** **(sustainability)**																	
	**Everyday mindfulness**										6.20 (2, 72)			0.147			.*003*
		App (n=21)^b^	1.48 (1.08)		1.33 (0.73)		1.95 (1.50)										
		App+O (n=24)^c^	2.88 (1.42)		2.42 (1.44)		2.42 (1.59)										
		App+OPS (n=30)^c^	2.33 (1.37)		2.87 (1.59)		2.73 (1.70)										
	**Future app (Headspace) use**										0.11 (2, 72)			0.003			.89
		App (n=21)	3.33 (1.24)		3.19 (1.29)		2.67 (1.32)										
		App+O (n=23)	3.43 (1.20)		3.00 (1.41)		2.78 (1.38)										
		App+OPS (n=31)	3.68 (0.98)		3.32 (1.14)		2.55 (1.09)										
	**Future mindful practice on own**										3.10 (2, 73)			0.078			.05
		App (n=21)^b^	2.81 (1.33)		2.86 (1.35)		3.00 (1.34)										
		App+O (n=24)^c^	3.71 (1.08)		3.75 (1.15)		3.50 (1.29)										
		App+OPS (n=31)^b,c^	3.16 (1.16)		3.39 (1.05)		3.32 (1.30)										
	**Future mindfulness in life**										7.42 (2, 71)			0.173			.*001*
		App (n=21)^b^	3.10 (1.18)		3.14 (1.35)		2.86 (1.46)										
		App+O (n=24)^c^	4.13 (0.85)		3.96 (1.08)		4.04 (1.23)										
		App+OPS (n=29)^c^	4.03 (0.82)		4.00 (0.85)		3.66 (1.08)										
**Skill learning (perceived benefits)**																	
	**Learned about mindfulness**										6.02 (2, 73)			0.142			.*004*
		App (n=21)^b^	3.33 (0.97)		3.57 (1.17)		3.62 (1.02)										
		App+O (n=24)^c^	4.04 (0.75)		4.00 (0.98)		4.29 (0.75)										
		App+OPS (n=31)^c^	4.06 (0.73)		4.19 (0.65)		4.10 (0.70)										
	**Learned mindfulness skills**										11.01 (2, 72)			0.234			<.*001*
		App (n=21)^b^	3.24 (1.09)		3.43 (1.21)		3.67 (0.91)										
		App+O (n=24)^c^	4.17 (0.70)		4.29 (0.75)		4.29 (0.86)										
		App+OPS (n=30)^c^	4.13 (0.63)		4.33 (0.55)		4.17 (0.70)										
	**Awareness of thoughts and feelings**										6.05 (2, 73)			0.142			.*004*
		App (n=21)^b^	3.10 (1.18)		3.33 (1.24)		3.52 (1.17)										
		App+O (n=24)^c^	4.04 (0.75)		3.96 (0.81)		4.17 (0.70)										
		App+OPS (n=31)^c^	3.97 (0.84)		4.06 (0.81)		3.84 (0.86)										

^a^We evaluated the skewness of the data for the 9 outcome variables at each of the 3 time points across the whole sample (Multimedia Appendix 2). Only 11% (3/27) of the variables exceeded thresholds (−1 to 1) for symmetry of the normal distribution. The skewed distribution for these 3 variables is not anticipated to increase the risk for type I errors [52]. Of note, the distribution of these 3 variables were not skewed at the midpoint. Rather, the distribution of these variables became skewed over time. This data pattern indicates a reduction in app use over time, rather than an unexpected pattern of behavior. The Kolmogorov-Smirnov tests were significant (*P*<.05; Multimedia Appendix 2), indicating that the residuals were not normally distributed. However, the central limit theorem suggests that for larger sample sizes (≥100), the violation of normality does not present a significant concern. Furthermore, the sampling distribution of the observations may be normal, even if the residuals do not precisely follow a normal distribution. For partial eta squared, ηp2≥0.01 indicates a small effect, ηp2≥0.06 indicates a medium effect, and ηp2≥0.14 indicates a large effect. *P* values in italics meet the criteria for the Bonferroni-adjusted significance threshold (*P*<.0055).

^b,c^Pairwise contrasts with different footnote designators are significant after Bonferroni adjustment for multiple comparisons.

Similarly, there was an overall effect of group (human support condition) on cumulative any and all (mindfulness and nonmindfulness) sessions, *F*_2,117_=15.00, η_p_^2^=0.20; *P*=.001. Pairwise comparisons indicate that App participants completed significantly fewer total sessions than App+O (*P*=.001) and App+OPS (*P*<.001) participants, but total sessions did not significantly differ between App+O and App+OPS groups (*P*=.37; Figure 2B).

#### Practice Integration (Sustainability)

As shown in Table 2 (with means and SDs), there was an overall effect of group (human support condition) on self-reported everyday nonguided mindfulness practice, *F*_2,72_=6.20, η_p_^2^=0.15; *P*=.003. Pairwise comparisons, with conservative Bonferroni correction, indicate that App participants engaged in significantly less nonguided mindfulness practice than App+O (*P*=.02) and App+OPS (*P*=.005) participants, but everyday nonguided mindfulness practice did not significantly differ between App+O and App+OPS groups (*P*=.99; Figure 3A).

There was no effect of group (human support condition) on self-reported likelihood to use the Headspace app in the future (*F*_2,72_=0.11, η_p_^2^=0.003; *P*=.89; Figure 3B). Meanwhile, the effect of group on self-reported likelihood to practice mindfulness exercises on one’s own in the future was right at the cutoff of statistical significance, with a medium effect size (*F*_2,73_=3.10, η_p_^2^=0.08; *P*=.05). Pairwise comparisons indicate that App participants were significantly less likely to believe that they would engage in mindfulness exercises on their own in the future compared to App+O (*P*=.045) participants. App+OPS participants did not differ from App (*P*=.51) or App+O (*P*=.60) participants (Figure 3C).

There was an overall effect of group (human support condition) on self-reported likelihood of future mindfulness in everyday life (*F*_2,71_=7.42, η_p_^2^=0.17; *P*=.001). Pairwise comparisons indicate that App participants were less likely to believe that they would engage in everyday mindfulness in the future than App+O (*P*=.002) and App+OPS (*P*=.006) participants. App+O and App+OPS groups did not differ (*P*=.99; Figure 3D).

#### Skill Learning (Perceived Benefits)

As shown in Table 2, there was an overall effect of group (human support condition) on the extent to which participants reported that they had learned about mindfulness through the intervention (*F*_2,73_=6.02, η_p_^2^=0.14; *P*=.004). Pairwise comparisons indicate that App participants endorsed learning less about mindfulness than App+O (*P*=.01) and App+OPS (*P*=.007) participants, and App+O and App+OPS groups did not differ (*P*=.99; Figure 4A).

Similarly, there was an overall effect of group (human support condition) on the extent to which participants reported that they had gained mindfulness skills through this intervention (*F*_2,72_=11.01, η_p_^2^=0.23; *P*<.001). App participants endorsed gaining mindfulness skills to a lesser extent than App+O (*P*<.001) and App+OPS (*P*<.001) participants, and App+O and App+OPS groups did not differ (*P*=.99; Figure 4B).

Finally, there was also an overall effect of group (human support condition) on the extent to which participants reported that they had gained awareness of their thoughts and feelings through this intervention (*F*_2,73_=6.05, η_p_^2^=0.14; *P*=.004). App participants endorsed gaining this awareness to a lesser extent than App+O (*P*=.006) and App+OPS (*P*=.01) participants, and App+O and App+OPS groups did not differ (*P*=.99; Figure 4C).

## Discussion

### Summary of Findings

This study demonstrated that empirically driven human support enhancements can increase (1) engagement with an mTDI; (2) the integration of intervention practice into daily life; and (3) learning, including perceived intervention-based awareness and skills. Specifically, this study found that users randomly allocated to attend a 90-minute, face-to-face orientation session to the mTDI—whether with or without additional random allocation to a peer supportive accountability group—demonstrated significantly higher (1) intervention engagement, including total minutes of mindfulness content and total number of sessions overall; (2) integration of intervention practice into everyday life, both currently and anticipated in the future; and (3) learning about mindfulness and related skills and an awareness of thoughts and feelings. Among participants randomly allocated to attend an orientation, those who were further randomly allocated to peer supportive accountability groups exhibited higher mean scores on app use across the trial assessment points, but this was not a significant difference. Furthermore, participants randomly allocated to either of the human support–enhanced conditions generally demonstrated significant benefits for the integration of skills and perceived learning compared to those randomly allocated to use the app alone, but there were no significant differences between these 2 human support–enhanced groups. These findings highlight the varied benefits of including human support in otherwise self-guided digital mental health interventions, but they leave open important questions about the connections among human support, engagement, and broader benefits for daily-life integration and learning.

### mTDI Engagement, Practice Integration, and Skill Learning: Understanding and Optimizing Connections

#### Overview

This study builds on the extensive evidence base demonstrating that mTDIs, specifically mindfulness-based mTDIs, have numerous benefits [1-3] and contributes to emerging research on how best to harness these benefits, both for increased engagement with the mTDI and for extended benefits into daily life. This study demonstrated broad benefits for human support on in-app engagement and real-life learning and applications of the intervention skills. These findings help advance our understanding of mTDI implementation science; yet, they also point to the need for continued research to better understand—as described in the following subsections—(1) the *active ingredients* of human support enhancements, including (2) *supportive accountability* and (3) the *optimal dosage* or level of mTDI engagement.

#### Active Ingredients of Human Support: What Promotes Success?

This study contributes to an emerging field of research attempting to better understand the active ingredients or mechanisms driving the benefits of human support. A recent review highlighted that including human support features that provide guidance (such as this study’s orientation session) and foster social connectedness (initiated in this study’s orientation session and deepened by the peer supportive accountability group) facilitate program engagement [15]. It is likely that human support serves, in part, to provide *reminders* for program engagement, which in turn enhance outcomes such as engagement, skill learning, and future intended practice [2]. However, it is notable that in this study, participants in the App+OPS group, who received frequent cues for mTDI engagement via the Facebook group, email digests, and in-person peer meetings, did not exhibit additive benefit beyond the orientation. These findings might indicate that receiving *personal, interactive guidance* at the outset was a critical ingredient for engaging with a new mTDI, similar to other research [53]. Navigating the initial setup of an mTDI and exploring its content can be overwhelming for users, especially when lacking guidance and feedback [54]. Similarly, receiving this guidance from research staff early on may have enhanced the *credibility* of the mTDI, which has been linked to greater interest in downloading and using the mTDI [55] as well as continued engagement over time [56]. Having our team of trained interventionists conduct the orientation session may have been particularly impactful on building credibility because the mTDI may have been viewed as having expert endorsement [55].

In line with prior evidence on the benefits of brief motivational enhancement interventions for mTDI engagement [35-37], it is also plausible that the *content* of the orientation helped promote participant engagement with the mTDI. Overall, this study adds support for the utility of offering participants an initial orientation session to provide psychoeducation about the skills incorporated into the mTDI, build familiarity with the mTDI, provide guidance related to engagement, and foster motivation and connection. This represents an important step in examining and comparing the effects of different components of human support, although future research is needed to isolate the specific active ingredients of the orientation, including credibility, personalized guidance, motivational enhancement, and proactive efforts to address challenges and barriers. Within the larger field of digital mental health, it is increasingly recognized that human support features vary widely across studies, and few adequately describe what exactly these features entailed [22]. As a result, the effects of such enhancements have been mixed, with some studies finding benefits and others not, as reviewed by Bernstein et al [22]. Clarifying the impact of human support and the active ingredients of such support will also inform the answers to questions such as who should provide support (eg, peer, paraprofessional, or professional), when it should be provided (eg, once at the beginning or interspersed throughout the intervention), and the content or focus of the support (eg, providing psychoeducation, offering encouragement, evoking motivation, or helping with problem-solving). Ultimately, the answers to such questions are likely to be complex and will require future research exploring how individual differences interact with support enhancements.

#### The Impact of Supportive Accountability on mTDI Engagement

Although participants in the App+OPS group used the app more than those in the App+O group, this was not a significant difference. This finding suggests little additional benefit of the peer supportive accountability group above and beyond the orientation alone. However, there are many considerations when interpreting this finding. First, it is possible that the App+OPS group may have experienced other, intangible benefits beyond impact on objectively captured mTDI engagement; for example, participants in the App+OPS group overwhelmingly indicated that they enjoyed connecting with other students over shared experiences and generally found the group to be helpful and supportive [57]. This corroborates prior findings that peer supportive accountability is typically viewed positively by participants [24,29,41]. Second, the orientation allowed for some interaction with peers, including fellow participants as well as student members of the research team; therefore, App+O participants may have experienced some degree of peer supportive accountability as well. This study design is not fully sufficient to tease apart the impact of peer supportive accountability alone, and more research is needed on how best to harness its potential benefits.

Third, this study’s findings may reflect the utility of integrating supportive accountability from a trained team of clinical *researchers*, without the additional benefit of adding supportive accountability from *peers*. Although all intervention groups, including those who used the mTDI without an orientation session (App group), had some degree of researcher contact across the trial (eg, for study consenting and assessment administration), the orientation session may have fostered a greater sense of participant accountability to the clinically trained research team, who facilitated the orientation. This is in line with reviews of mTDIs generally, which find that participants who enroll in mTDIs without any research contact show significantly higher attrition rates over time compared to participants who enroll through contact with research staff [13]. Those users who get started without researcher contact may not fully appreciate the effort and internal motivation that will be required to begin and sustain engagement over time [13], whereas researchers may be able to provide a more realistic picture to participants who do have contact with them. Therefore, it is critical to consider how human support enhancements may be implemented beyond the research context. Engagement with mTDIs is significantly improved by the structure and containment of research trials compared to engagement with the same programs in the “real world” [11]; therefore, finding ways to translate the benefits of an orientation session to the everyday mTDI user will be important.

This study’s findings also raise questions about when, as well as how, to integrate human support. Our findings suggest that initial support (eg, an orientation) as users are beginning a new mTDI may be more beneficial for sustained engagement than continued support over time. Furthermore, with high levels of human support throughout the intervention, users could become reliant on external motivation and then decrease or stop engagement when the support is removed. In this study, the App+OPS group experienced a stark drop in human support, and likely supportive accountability, at the 2-month mark when the meetings ended and they stopped receiving email digests and posts in the web-based forum. Although app use tapered in all groups during the follow-up period, the decline was visually steepest for the App+OPS group. Though the pairwise contrasts between the App and App+O groups were not statistically significant, this pattern is worth investigating in future research. Perhaps, there is an optimal level of human support that, when tapered gradually, is conducive to sustaining mTDI engagement over the longer term.

#### Optimal Dosage of mTDI Engagement

Although certainly some level of engagement is needed to see benefits, the *optimal dosage* of mTDI use remains an open question. Research has recently begun to move beyond investigating the overall efficacy of mTDIs and toward exploring the dose-response relationship between mTDI engagement and outcomes [58]. Often, there is an implicit assumption that more is better, but there is little understanding of the dose-response effect between mTDI engagement and outcomes and therefore the ideal amount of mTDI use [59,60]. More research is needed to better understand the complex relations between use and treatment response to promote optimal engagement and benefits.

There also remain questions about the reasons driving drop-offs in engagement after initial periods of use [61,62]. Declining mTDI use over time may not necessarily be indicative of a user’s lack of interest in a program, lack of progress, or symptom severity. On the contrary, some users may have internalized the skills learned through the app or otherwise met their goals (eg, behavioral change and symptom improvement) such that they no longer needed to rely on the mTDI for support. Similar to attending psychotherapy sessions, mTDIs can be viewed as training wheels that provide users with psychoeducation and skill learning and practice. This, in turn, allows users to create personal well-being habits beyond the intervention, such as practicing mindfulness or other intervention skills in daily life—outside of an mTDI and beyond trial periods—as evidenced in this study. Thus, in some cases, the dwindling use of an mTDI after initial engagement might, in fact, be a marker of success. At a minimum, relying only on objectively measured app use through mTDI user data likely does not capture the full scope of individuals’ engagement with the intervention content. Similar to this study, others have begun to incorporate additional self-report measures of skills practice outside of mTDIs and found that this is an important element in predicting benefits [60,63].

### Limitations and Future Directions

This study used a rigorous RCT design with intent-to-treat analyses to examine multiple indicators of mTDI engagement and benefits, including objectively measured app use and self-reported practice integration and skill learning, through a 1-month follow-up period; yet, there are important limitations to address as well. First, although an RCT is the gold standard for establishing general intervention effects, other research is important for evaluating diverse intervention benefits (eg, dose-response effects, clinical utility, and social validity) as well as revealing processes and mechanisms of change. Future research should continue to explore various designs with a broader array of outcomes and more diverse samples to improve generalizability. Second, it is important to note that the onset of the COVID-19 pandemic occurred during the sixth semester of data collection, necessitating changes (eg, remote data collection, orientation, and App+OPS meetings) impacting cohorts 6 to 8. Third, although mTDIs provide methodological benefits by tracking in-app engagement and skills practice, data recording errors can occur; for example, 2.4% (3/123) of participants were excluded from mTDI engagement analyses due to glitches with their app-recorded use data. It is possible that other data-tracking errors could have occurred without the researchers’ knowledge. Similarly, it is possible that participants could be inattentive, distracted, or asleep during some of the practices recorded by the app. Fourth, although the broad recruitment strategies and voluntary response sampling method have benefits for generalizability, they also yielded a sample consisting largely of women (reflecting both the higher proportion of women in college and their greater likelihood of meeting criteria for depression), which limits generalizability in other ways. Relatedly, not all participants completed the surveys assessing self-reported practice integration and skill learning, further limiting the generalizability of the findings.

Fifth, it is important to note that the App condition included participants who activated their Headspace access code at the same time as the rest of the cohort as well as a subset of participants who accessed their code *after* a 3-month waitlist period. Although analyses did not reveal any significant differences in mental health symptoms between App participants who could access Headspace immediately and those who waited 3 months, it is possible that combining these 2 groups may have affected the results of the App condition, particularly because prior research suggests that intervention engagement can be negatively impacted by time spent waiting for services [64].

Sixth, and related, the 3 randomly allocated groups were nested such that participants in all 3 groups received the base intervention (App), 2 of the nested groups attended an orientation (App+O and App+OPS), and 1 of these nested groups included additional peer supportive accountability features (App+OPS). Analyses tested for the incremental benefits of the 2 human support enhancements; yet, future research might benefit from examining the benefits of an app+peer supportive accountability condition without an orientation session.

Finally, this study’s implementation of peer supportive accountability had some limitations that may have reduced the potential impact of the group on mTDI engagement and the integration of skills. The web-based forum included a “leaderboard” feature that displayed each member’s use of the app from the current week and the trial period overall. This may have promoted external motivation, which is typically not as effective as intrinsic motivation for sustained engagement [15]. This type of feature also may function differently for individuals experiencing depression compared to those with other mental health symptoms. Given that self-criticism and negative self-concept are common among individuals with depression [65,66], having progress metrics shared with the larger group may have been perceived as discouraging rather than motivating. In addition, the use of Facebook for the online forum—selected for its social connection and privacy features—may have made engagement with the peer group more difficult because Facebook is becoming increasingly less popular among young adult participants [67]. Future studies should explore alternative platforms for web-based interaction among participants, ideally integrating all peer support features into the mTDI. Furthermore, future research should explore whether the benefits of human support, such as those demonstrated in this study, might also be harnessed with automated support, whether preprogrammed or assisted by interactive artificial intelligence. Beyond the more readily automatized elements such as providing information and reminders, research on the benefits of human support tends to emphasize more complex elements such as personalized prompts, interactive coaching, and professional guidance [2,22,34,53,54,68]. Notably, recent advances in artificial intelligence and machine learning might allow for the transfer of some human support elements and benefits to automated programming [2]. Importantly, these developments raise crucial ethical and societal concerns that must be addressed [69].

### Clinical Applications and Research Implications

This study’s findings have important implications that move beyond foundational research on *whether* to use mindfulness-based mobile apps in clinical intervention work by investigating *how* best to maximize their benefits, such as through brief, face-to-face orientations and through small-group peer support networks. These results can be applied more broadly to mTDIs and also are likely to extend to other user populations; for example, the peer supportive accountability model implemented here—with the notable request for more face-to-face contact by the participants themselves—might be similarly relevant for mTDI users who share a common identity or life circumstance, such as high school students, employees, veterans, parents, or patients [25,26,28].

Boosting mTDIs with human support features has numerous applications in various settings and with diverse people who can serve to support engagement and thus enhance benefits; for example, many health care systems allow for providers within different departments (eg, behavioral health, primary care, and specialty clinics) to refer patients to mTDIs as a means of addressing lower-severity symptoms, supplementing preexisting mental health services, or bridging the patient to other levels of care, such as while they are on a waitlist [70]. Similarly, many employers now offer mental health mTDIs as part of their health insurance package [71], and there is emerging research on integrating mental health care into routine curricular and extracurricular settings on college campuses [72-74]. While mTDIs are increasingly incorporated into nontraditional settings, the typical approach is simply to provide individuals with access to the mTDI, leaving users to download and engage with the program on their own. The findings from this study suggest that evidence-based mTDIs can be even more effective and have a more powerful reach and impact if supplemented with human support; for example, professionals, paraprofessionals (without a mental health degree), or peers can serve as coaches to support engagement with evidence-based mTDIs through routine settings such as primary care clinics [75], workplaces [53], and college student academic services [76]. This study also suggests that an initial, one-time group introduction to the mTDI conducted by a knowledgeable source can produce a myriad of benefits and represents a cost-effective and scalable option for larger institutions; for example, hospitals and clinics, schools and community agencies, and corporate and nonprofit organizations can pair their offerings of mTDIs with regularly occurring orientation sessions that assist new users in accessing the program, demonstrate the app and its features, answer questions, and elicit motivation. This would allow many potential users to join at once, thus improving efficiency, and might also boost social connection and a sense of community—which in turn would promote mTDI use and well-being more broadly.

### Conclusions

While mTDIs have great promise for increasing access to well-being interventions, relatively low rates of user uptake and engagement are current barriers to achieving the potential reach and impact of mTDIs. The results of this study illustrate that human support—particularly an initial face-to-face orientation session that provides psychoeducation, builds familiarity with the mTDI, and promotes motivation and connection—boosts mTDI engagement, enhances the integration of intervention practice into everyday life, and increases skill learning. Future work is needed to determine the active ingredients of the orientation (eg, credibility, personalized guidance, motivational enhancement, and proactive problem-solving) that might drive increased levels of engagement and the associated positive intervention benefits. Identifying optimal intervention dosage levels for evidence-based mTDIs also remains critical to fully harnessing the potential of these interventions and their role in improving societal health and well-being.

## Data Availability

The datasets generated and analyzed during this study are available from the corresponding author on reasonable request.

## Appendix

Multimedia Appendix 1 CONSORT-EHEALTH (Consolidated Standards of Reporting Trials of Electronic and Mobile Health Applications and Online Telehealth) checklist (V 1.6.1).

Multimedia Appendix 2 Skewness values and SEs from the Kolmogorov-Smirnov test with Lilliefors correction for study outcome variables at 3 time points for the full study sample.

## Abbreviations

App: app as usual
App+O: app+orientation
App+OPS: app+orientation and peer supportive accountability
CONSORT: Consolidated Standards of Reporting Trials
MI: motivational interviewing
mTDI: mobile technology–delivered intervention
PHQ-8: Patient Health Questionnaire-8
RCT: randomized controlled trial
TDI: technology-delivered intervention
