# Supplementary material for: Enhancing Engagement, Practice Integration, and Skill Learning in Mobile Technology–Delivered Interventions Using Human Support: Randomized Controlled Trial With Depressed College Students
Source: JMIR Form Res. 2025 Aug 25;9:e56963. doi: 10.2196/56963 (PMC12417905; doi:10.2196/56963)
Supplement: Multimedia Appendix 2 [file formative_v9i1e56963_app2.pdf]

Multimedia Appendix 2

From: Conley, C.S., DeLorenzo, B.M., Gonzales, C.H., Kahrilas, I.J., Duffecy, J., & Silton, R.L. (2025). **Enhancing engagement, practice integration, and skill learning in mobile technology-delivered interventions using human support: Randomized controlled trial with depressed college students.** *JMIR Formative Research, e:56963.* https://doi.org/10.2196/56963

We evaluated the skewness of the data for the 9 outcome variables at each of the 3 time points across the whole sample. Only 11% (3/27) of the variables exceeded thresholds (−1 to 1) for symmetry of the normal distribution. The skewed distribution for these 3 variables is not anticipated to increase the risk for type I errors (Knief & Forstmeier, 2021). Of note, the distribution of these 3 variables were not skewed at the midpoint. Rather, the distribution of these variables became skewed over time. This data pattern indicates a reduction in app use over time, rather than an unexpected pattern of behavior. The Kolmogorov-Smirnov tests were significant ($P<.05$; Multimedia Appendix 2), indicating that the residuals were not normally distributed. However, the central limit theorem suggests that for larger sample sizes (≥100), the violation of normality does not present a significant concern. Furthermore, the sampling distribution of the observations may be normal, even if the residuals do not precisely follow a normal distribution.

Table S1.

Skewness Values and Standard Errors for Study Outcome Variables at Three Time Points, for the Full Study Sample.

**Outcome**	Mid (1mo) Skewness[SE]	Post (2mo) Skewness[SE]	Follow-Up (3mo) Skewness[SE]
**Engagement with mTDI**			
Cumulative Mindfulness Minutes	.756 (.240)	**1.025** (.239)	**3.972** (.240)
Cumulative Total Sessions	.425 (.240)	.772 (.239)	**3.735** (.240)
**Practice Integration [Sustainability]**			
Everyday Mindfulness	.665 (.222)	.657 (.223)	.645 (.257)
Future App (Headspace) Use	-.329 (.222)	.061 (.223)	.292 (.255)
Future Mindful Practice on Own	-.173 (.221)	-.360 (.223)	-.127 (.255)
Future Mindfulness in Life	-.669 (.222)	-.745 (.224)	-.387 (.255)
**Skill Learning [Perceived Benefits]**			
Learned About Mindfulness	-.178 (.241)	-.927 (.459)	-.677 (.287)

**Outcome**	Mid (1mo) Skewness[SE]	Post (2mo) Skewness[SE]	Follow-Up (3mo) Skewness[SE]
Learned Mindfulness Skills	-.880 (.241)	-.873 (.243)	-.594 (.289)
Awareness of Thoughts & Feelings	.048 (.243)	-.203 (.244)	-.330 (.287)

Notes. Three out of the 27 variables exceeded skewness thresholds (-1 to 1) for symmetry of the normal distribution.

Table S2.

Kolmogorov-Smirnov (K-S) Test with Lilliefors Correction at Three Time Points, for the Full Study Sample.

**Outcome**	Mid (1mo) Skewness	Post (2mo) Skewness[SE]	Follow-Up (3mo) Skewness[SE]
**Engagement with mTDI**			
Cumulative Mindfulness Minutes	.144*	.333*	.100*
Cumulative Total Sessions	.140*	.140*	.321*
**Practice Integration [Sustainability]**			
Everyday Mindfulness	.285*	.285*	.302*
Future App (Headspace) Use	.198*	.181*	.200*
Future Mindful Practice on Own	.175*	.190*	.182*
Future Mindfulness in Life	.262*	.238*	.193*
**Skill Learning [Perceived Benefits]**			
Learned About Mindfulness	.239*	.256*	.251*
Learned Mindfulness Skills	.271*	.253*	.233*
Awareness of Thoughts & Feelings	.222*	.197*	.233*

Notes. **p* <.05; Statistically significant results indicate that the null hypothesis should be rejected, indicating that the residuals do not follow the normal distribution.

Reference

Knief U, Forstmeier W. Violating the normality assumption may be the lesser of two evils. Behav Res Methods. Dec 07, 2021;53(6):2576-2590. [doi: 10.3758/s13428-021-01587-5] [Medline: 33963496]
